# Sex Hormones Promote Opposite Effects on ACE and ACE2 Activity, Hypertrophy and Cardiac Contractility in Spontaneously Hypertensive Rats

**DOI:** 10.1371/journal.pone.0127515

**Published:** 2015-05-26

**Authors:** P. L. M. Dalpiaz, A. Z. Lamas, I. F. Caliman, E. H. Ribeiro, G. R. Abreu, M. R. Moyses, T. U. Andrade, S. A. Gouvea, M. F. Alves, A. K. Carmona, N. S. Bissoli

**Affiliations:** 1 Department of Physiological Sciences, Federal University of Espirito Santo, Vitória, Espirito Santo, Brazil; 2 Department of Pharmacy, University Vila Velha, Vila Velha, Espirito Santo, Brazil; 3 Nucleus of Biotechnology, Federal University of Espirito Santo, Vitória, Espirito Santo, Brazil; 4 Department of Biophysics, Escola Paulista de Medicina, Federal University of São Paulo, São Paulo, Brazil; Max-Delbrück Center for Molecular Medicine (MDC), GERMANY

## Abstract

**Background:**

There is growing interest in sex differences and RAS components. However, whether gender influences cardiac angiotensin I-converting enzyme (ACE) and angiotensin-converting enzyme 2 (ACE2) activity is still unknown. In the present work, we determined the relationship between ACE and ACE2 activity, left ventricular function and gender in spontaneously hypertensive rats (SHRs).

**Methodology / Principal Findings:**

Twelve-week-old female (F) and male (M) SHRs were divided into 2 experimental groups (n = 7 in each group): sham (S) and gonadectomized (G). Fifty days after gonadectomy, we measured positive and negative first derivatives (dP/dt maximum left ventricle (LV) and dP/dt minimum LV, respectively), hypertrophy (morphometric analysis) and ACE and ACE2 catalytic activity (fluorimetrically). Expression of calcium handling proteins was measured by western blot. Male rats exhibited higher cardiac ACE and ACE2 activity as well as hypertrophy compared to female rats. Orchiectomy decreased the activity of these enzymes and hypertrophy, while ovariectomy increased hypertrophy and ACE2, but did not change ACE activity. For cardiac function, the male sham group had a lower +dP/dt than the female sham group. After gonadectomy, the +dP/dt increased in males and reduced in females. The male sham group had a lower -dP/dt than the female group. After gonadectomy, the -dP/dt increased in the male and decreased in the female groups when compared to the sham group. No difference was observed among the groups in SERCA2a protein expression. Gonadectomy increased protein expression of PLB (phospholamban) and the PLB to SERCA2a ratio in female rats, but did not change in male rats.

**Conclusion:**

Ovariectomy leads to increased cardiac hypertrophy, ACE2 activity, PLB expression and PLB to SERCA2a ratio, and worsening of hemodynamic variables, whereas in males the removal of testosterone has the opposite effects on RAS components.

## Introduction

The renin-angiotensin system (RAS) is a complex, mixed enzymatic-hormonal system that controls electrolyte balance, blood volume and arterial blood pressure [1]. RAS activation is also essential for structural and functional heart remodelling [2]. The “classical” RAS pathway consists of interactions between angiotensin I (Ang I), angiotensin II type 1 (AT_1_) receptors [3] and two main enzymes, renin and angiotensin converting enzyme (ACE) [1], that act in series to liberate the active octapeptide angiotensin II (Ang II). Ang II is responsible for all of the peripheral effects associated with the classical system. Among these classical effects are arterial pressure regulation and cardiac and vascular function, including vasoconstriction, sodium reabsorption, cell proliferation, and vascular hypertrophy [4]. The non-classical RAS pathway consists of ACE2, angiotensin 1–7 (Ang 1–7), the Ang II receptor AT2, and Mas receptors that oppose AT_1_-mediated effects and cause vasodilation, improve blood flow, and enhance pressure-natriuresis [5,6].

Studies in humans and hypertensive animal models have demonstrated the importance of the interaction between sex hormones and RAS in regulating cardiovascular function and blood pressure [7–9]. Much evidence supports that males have greater expression of “classical” RAS components Ang II and AT_1_, whereas females have greater expression of “non-classical” RAS components such as AT2 and Ang (1–7) [10]. Additionally, we [11] and others [8,12] have demonstrated differences in the effect of ACE inhibitors between males and females.

According to some authors, sex hormones may be a key factor in the observed differences in the rates and severity of cardiac diseases between genders [9,13]; however, the exact mechanisms by which sex hormones contribute to this regulation are still unclear. We hypothesised that in hypertensive animals, these mechanisms could involve the interaction between sex hormones and cardiac RAS components. Therefore, the present study was designed to investigate whether: 1) there are sex differences in cardiac ACE and ACE2 activity in SHRs; 2) there are sex differences in hemodynamic variables in SHRs, and 3) if these variables are influenced by alterations in hormonal status.

## Materials and Methods

### Animals

The study was conducted in 12-week-old male and female SHRs that initially weighed 165 ± 7 g (females) and 243 ± 6 g (males) and had free access to food and water. The rats were divided into 2 experimental groups (n = 7 in each group) of female (F) and male (M) rats as follows: sham-operated (S) and gonadectomized (G). The rat systolic blood pressure was measured at the onset of the experiment and after 7 weeks (50 days after gonadectomy) by plethysmography. Body weight was also monitored during the experiment. The animals were housed in standard plastic cages at a constant temperature of 22°C and exposed to a 12-hour light-dark cycle. All of the experimental protocols were performed in the same animals in both groups. The procedures were performed in compliance with the guidelines for the ethical use of animals in scientific research and were approved by the Ethical Committee of the Federal University of Espírito Santo (Espírito Santo, Brazil). All of the surgical procedures were performed under ketamine (70 mg/kg i.p.) and xylazine (10 mg/kg i.p.) anesthesia.

### Experimental Design

#### Gonadectomy

Female rats were subjected to a skin incision of 1 to 1.5 cm and a muscular incision to open the peritoneal cavity for uterine tubules connection and ovary removal. Sham-operated rats underwent the same procedure, except the ovaries were exteriorized but not removed. The peritoneal cavity was then cleaned and sutured. The sham rat estrous cycle was monitored continuously with vaginal smears. This group was subjected to the experimental protocol on the day of proestrus. Orchiectomy was performed via an anterior median incision in the scrotum, and each testicle was exposed through the surgical incision. The ductus deferens was isolated, ligated, and severed, thus allowing the testicle to be removed. The incision was then closed and sutured with 3–0 chromic catgut.

### Determining estrous cycle phase

Daily vaginal smears were taken from each females rats as previously described [14] to confirm that their estrous cycles were proceeding normally. In the both groups, sham and ovariectomized rats, carefully, vaginal epithelial cells were collected and examined by the same experimenter, in the same period of the day (8:00 and 10:00 am), for up to 7 consecutive days before the experiment. Although there is no equivalent manipulation in males rats, these animals were paralleled handling to prevent the possible stress response-influence in the results. Females exhibiting normal estrous cycles were killed at proestrus between 9:00 am and 1:00 pm. After the experiment, all of the females had their uterus removed and weighed.

### Blood Pressure Determination

Systolic blood pressure (SBP) in conscious rats was measured using a tail-cuff manometer that was manufactured by IITC Life Science Inc. (Woodland Hills, CA, USA). The animals were placed inside a warming chamber (approximately 34°C) for 30 min before the measurements. The procedural aim was to calm the animals and dilate the tail blood vessels. The arterial blood pressure of each animal was measured at least three times, and any changes in pressure were reported as baseline value variation.

### Histological analysis

At the end of the experiment, animals were euthanized by decapitation, the hearts were excised, and the left ventricle (LV) was dissected, measured and normalized by tibial length (TL). We used the LV / TL and not LV / BW ratio because body weight changes with age in the SHR. As a result, the cardiac hypertrophy is quantified with greater accuracy with the LV/TL index [15]. Next, the ventricles were fixed in a 10% neutral buffered formalin solution for 24 hours followed by extraction of a mid-coronal ventricular tissue section and paraffin embedding. For cellular structure visualization, a 5-μm thick slice was stained with hematoxylin and eosin (H&E). Photographs of the samples were taken using an image acquisition system (Moticam Plus; Motic Inc., Canada). Morphometric analysis was performed by counting myocyte nuclei number per high power field and cardiac fiber diameter under 400X magnification. These analyses were performed using Moticam Plus histological analysis software. For each sample, 10 high-power fields were evaluated.

#### Cardiac ACE activity

ACE activity was determined in heart tissue and serum using the fluorescence resonance energy transfer (FRET) peptide Abz-FRK(Dnp)P-OH (Aminotech Pesquisa e Desenvolvimento, SP, Brazil) as a substrate [16]. Heart samples were quickly harvested, homogenized in 0.1 M Tris-HCl buffer, pH 7.0 containing 50 mM NaCl and centrifuged at 1000 g for 10 min. The assays were performed at 37°C in 0.1 M Tris-HCl buffer, pH 7.0 containing 50 mM NaCl and 10 μM ZnCl2. The hydrolysis rate of the substrate Abz- FRK(Dnp)P-OH (10 μM) after incubation for 30 min at 37°C with heart homogenates and serum aliquots was assessed to obtain ACE enzymatic activity. The assay methodology was adapted to a 96-well plate reader and the fluorescence was measured at 320 nm excitation and 420 nm emission in a Hitachi F-7000 spectrofluorimeter. Heart ACE activity was expressed in arbitrary fluorescence units (AFU/μg.prot.). The protein content was determined by the Lowry methods.

#### Cardiac ACE2 activity

Heart tissue ACE2 activity was determined using the 10 μM of the fluorogenic substrate Mca-APK(Dnp)-OH (Aminotech Pesquisa e Desenvolvimento, SP, Brazil) in 0.2 M Tris-HCl buffer, 200 mM NaCl, and 10 mM ZnCl2, pH 7.5, [17]. The increase in fluorescence was followed in a 96-well fluorescence plate reader Hitachi F-7000 (λ excitation = 320 nm; λ emission = 420 nm). Measurements were performed in duplicate, and ACE2 activity values were obtained as fluorescence arbitrary units (AFU). Assay specificity was demonstrated by hydrolysis inhibition with 10 μM DX600 (Phoenix Pharmaceuticals, Burlingame, CA), a specific ACE2 inhibitor.

### Hemodynamic Evaluation

Another set of rats was used for hemodynamic evaluation, the animals were anesthetized with ketamine (100 mg.kg-1, i.p., Agener União, Brazil) and xylazine (10 mg.kg-1, ip, Bayer, Brazil) the LV function of animals from sham operated and gonadectomized groups was assessed as previously described [18]. Briefly, the right common carotid artery was separated from connective tissue and catheterized with a fluid-filled polyethylene catheter (P50). The catheter was connected to a pressure transducer (TRI 21, Letica Scientific Instruments, Spain) and then to a digital system (Powerlab/4SP ML750, ADInstruments, Australia). After a 15 min stabilization period, the arterial systolic and diastolic blood pressure were recorded. The catheter was then advanced to the left ventricle. For an additional 15 min stabilisation period, functional variables were measured as LV dP/dt max, which is the maximum rate of ventricular pressure increase or the peak positive value of the first derivative of the left ventricular pressure, as well as the rate of pressure decay (-dP/dt). The signal was expressed in mmHg/sec. Following this procedure, the catheter was withdrawn from the LV and the arterial pressure was measured again to determine if damage to the aortic valve had occurred. The animal was not included if a decrease in the diastolic blood pressure was observed. Data were analysed using the LabChart software7.

### Western Blot Analysis

Western blot was performed as previously described [19]. Proteins from the homogenized left ventricle (25 mg for PLB and 80 mg for SERCA2a) were separated by 7.5% and 10% SDS- PAGE. Proteins were transferred onto nitrocellulose membranes, which were incubated with mouse monoclonal antibodies for SERCA2a (lot number: OF182464, catalog number of antibody: MA3-919; Molecular size 110, 1:1000, Affinity BioReagents, CO, USA) and PLB (lot number: OG185356, catalog number of antibody: MA3-922, Molecular size 25, 0.5 mg/ml, Affinity BioReagents, CO, USA). After washing, the membranes were incubated with anti-mouse (1:5000, Stressgen, Victoria, Canada) or anti- rabbit (1:7000, Stressgen, Victoria, Canada) immunoglobulin antibodies conjugated to horseradish peroxidase. After thorough washing, immunocomplexes were detected using an enhanced horseradish peroxidase/luminal chemiluminescence system (ECLPlus, Amersham International, Little Chalfont, UK) and film (Hyperfilm ECL International). Signals on the immunoblot were quantified with NIH Image J V1.56. Each membrane was reprobed to determine GAPDH expression using a mouse monoclonal antibody (1:5000, Abcam Cambridge, MA, USA).

### Statistical Analysis

The results for all of the groups are expressed as the mean ± standard error of the mean (SEM). Statistical evaluation was performed by repeated measures 2-way ANOVA followed by Fischer Least Significant Differences (LSD) post-hoc comparison. A P value <0.05 was considered to be statistically significant. The statistical analyses were performed with GB Stat software.

## Results

### Body, Heart and Uterus Weight

The morphological characteristics of male and female rats throughout the study are summarized in Table 1. Although male SHR were heavier than the females at both the start and the end of the experimental period, no significant differences were observed between animals within sex at both time points, except in the female group. After ovariectomy, females exhibited an increase in their final body weight when compared with the female sham group. As expected, the uterine weight/body weight (UW/BW) ratio and uterine weight/tibial length was decreased in ovariectomized females. The development of left ventricular hypertrophy was evaluated by LV weight/ tibial length ratios and histological variables. The LVW/TL ratio was higher in male rats compared to female rats. Castration reduced this parameter in males and increased it in female rats (Table 1).

**Table 1:** Effect of gonadectomy on body weight (BW), tibial length, uterine weight/body weight (UW/BW) ratio, left ventricle weigh (LVW) and weigh/tibial length ratio (LV/TL).

Group	Final BW	Tibial Length	U/BW	U/TL	Left Ventricle	LV/TL
n = 7	(g)	(cm)	(mg/g)	(mg/g)	(mg)	(mg/cm)
SM	278 ± 4*	3,8 ± 0,02*	___	___	763 ± 25*	200 ± 6*
SF	176 ± 2	3,6 ± 0,02	2,3 ± 0,10	0,627 ± 0,027	545 ± 4,5	150 ± 1
GM	287 ± 9*	3,8 ± 0,02*	___	___	697 ± 11*+	181 ± 3*+
GF	212 ± 2+	3,5 ± 0,04	0,41 ± 0,01+	0,112 ± 0,006 +	602 ± 6+	168 ± 2+

The results are the mean ± S.E.M (n = 6 per group). Variables measured in the Sham(S) and gonadectomized (G), male (M) and female (F) SHR rats. Statistical significance is indicated by *P, 0.05 vs. females of the same group

^+^P, 0.05 vs. sham vehicle animals of the same sex. (two-way ANOVA and Fischer test).

### Blood Pressure

**Fig 1:**
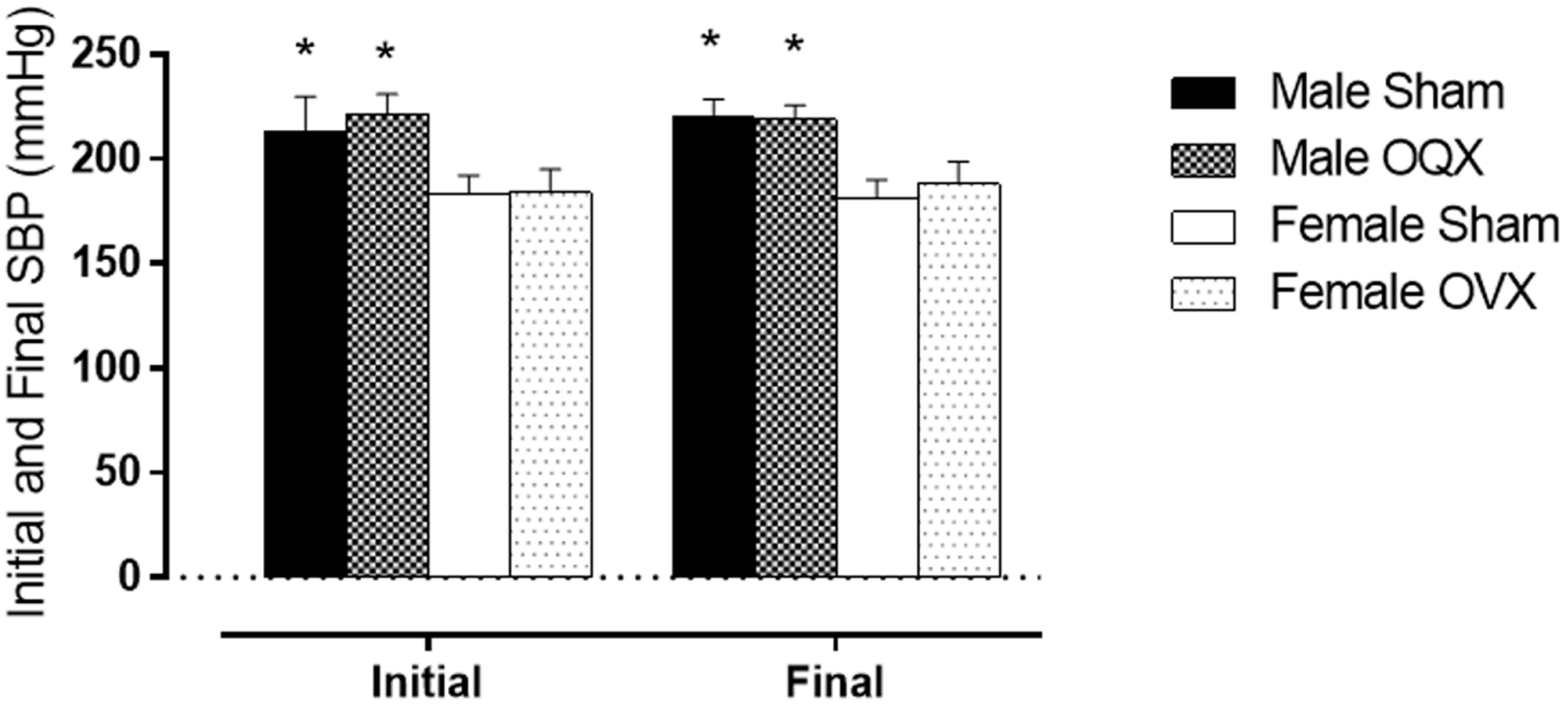
Initial and final systolic blood pressure. Male sham, male orchiectomized (OQX), female sham and female ovariectomized (OVX). The results are the mean ± S.E.M. (n = 7 per group). *P < 0.05 vs. females of the same group; (two-way ANOVA and Fischer test).

The Fig 1 shows the initial and final SBP of all of the groups studied. The initial SBP of the male (M) groups (MS = 213 ± 5 mmHg vs MG = 221 ± 3 mmHg) was higher than that of the female (F) groups (FS = 183 ± 3 mmHg vs FG = 184 ± 4 mmHg). After the experimental period, the final SBP of male rats (MS = 220 ± 2 mmHg vs MG = 219 ± 2 mmHg) continued to be higher than the SBP of female rats (FS = 181 ± 2 mmHg vs FG = 188 ± 2 mmHg). Gonadectomy (G) did not alter the blood pressure of either sex.

### Histological Analysis

**Fig 2:**
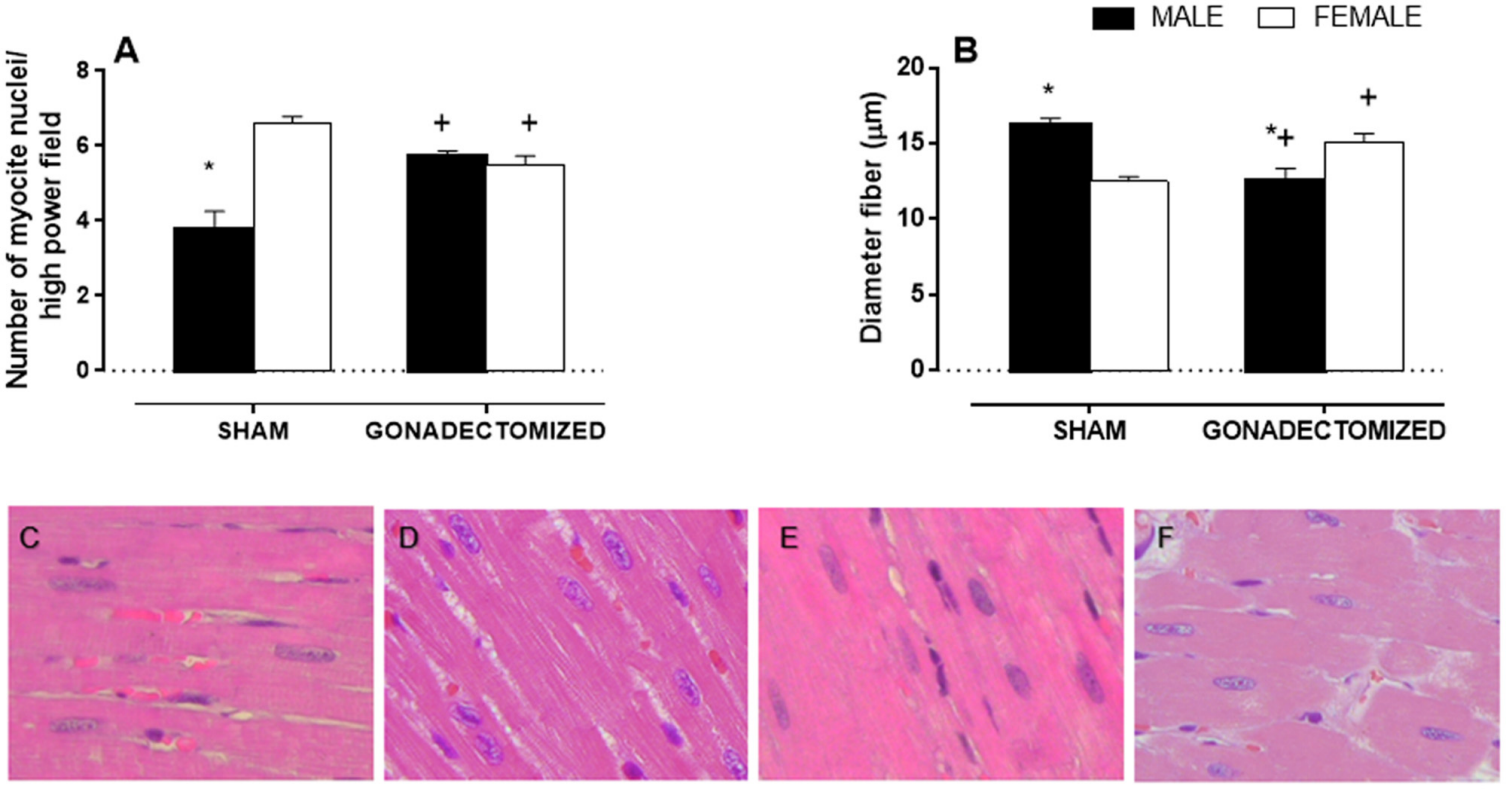
Histological analysis of the left ventricle in SHR. Sham and gonadectomized animals. A: number of myocyte nuclei/ high power field, B: cardiac fiber diameter. Representative images of histological analyses in C: sham male SHR, D: sham female groups, E: orchiectomized male groups and F: ovariectomized female groups. Samples were stained with hematoxylin/eosin (H&E): 400x. The results are the mean ± S.E.M. (n = 7 per group). Statistical significance is indicated by *P < 0.05 vs. females of the same group; ^+^P < 0.05 vs. sham animals of the same sex; (two-way ANOVA and Fischer test).

Fig 2 demonstrates the gender differences in nuclei number/high power field and cardiac fiber diameter. The male sham group had fewer nuclei than the female sham group (MS: 3,7 ± 0.5 vs. FS: 6,6 ± 0.4). Gonadectomy increased the number of nuclei in the males and reduced it in female rats (MG: 5,7 ± 0.1 vs. FG: 5,4 ± 0.6) compared with the sham group. Additionally, the male sham group had higher cardiac fiber diameter than the female sham group (MS: 16,1 ± 0,9 μm vs. FS: 12,5 ± 0,9 μm). Gonadectomy reduced cardiac fiber diameter in the males and increased it in the female rats (MG: 12,55 ± 0,8 μm vs. FG: 15,1 ± 0,9 μm) compared with the respective sham groups.

### Cardiac ACE and ACE2 Activity

**Fig 3:**
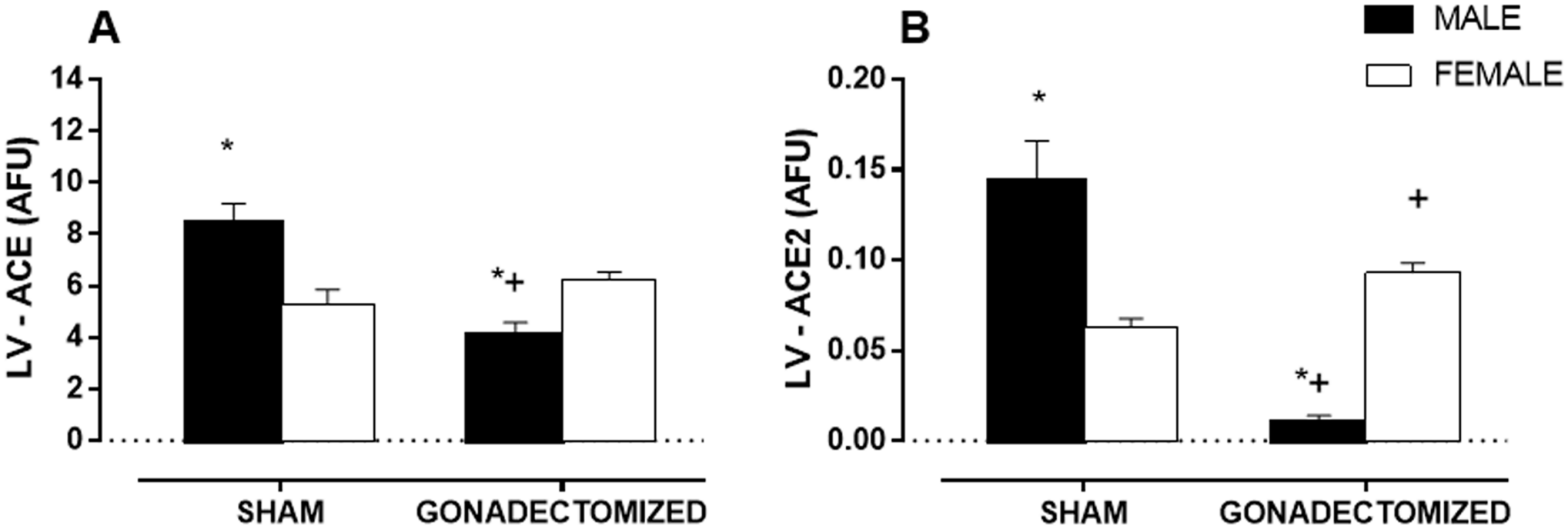
Left ventricle ACE and ACE2 activity. (A) ACE and (B) ACE2 activity values were obtained as fluorescence arbitrary units (AFU/μg.prot.) in male and female SHR, sham and gonadectomized groups. The results are the mean ± S.E.M. (n = 7 per group). Statistical significance is indicated by*P < 0.05 vs. females of the same group; ^+^P < 0.05 vs. sham animals of the same sex; (two-way ANOVA and Fischer test).

After the experimental period, we observed gender differences in ACE ventricular activity (MS: 8.5 ± 0.8 AFU vs. FS: 5.7 ± 0.5 AFU) and ACE2 (MS: 0.14 ± 0.002 AFU vs. FS: 0.06 ± 0.001 AFU); male rats exhibited higher values than the female rats (Fig 3A and 3B). Gonadectomy promoted opposite responses in the activity of both enzymes. The orchiectomy reduced the activity of ACE (MG: 4.0 ± 0.5 AFU) and ACE2 (MG: 0.01 ± 0.003 AFU), while ovariectomy increased the activity of ACE2 (FG: 0.09 ± 0.001 AFU) and did not change ACE activity (FG: 6.26 ± 0.8 AFU).

### Hemodynamic Evaluation

**Fig 4:**
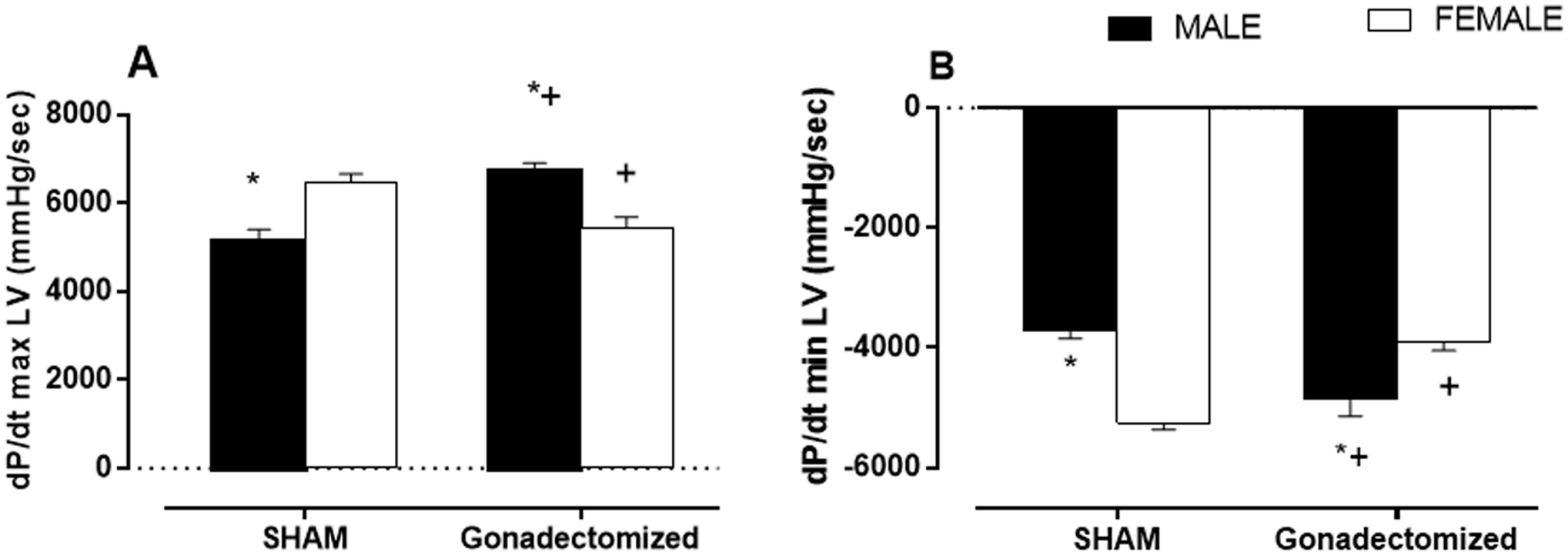
Hemodynamic variables. (A) positive first derivatives dP/dt maximum Left Ventricle (mmHg/sec) and (B) negative first derivatives dP/dt minimum (mmHg/sec) in male and female SHR, sham and gonadectomized groups. The results are the mean ± S.E.M. (n = 7 per group). *P < 0.05 vs. females of the same group; ^+^P < 0.05 vs. sham animals of the same sex; (two-way ANOVA and Fischer test).

Hemodynamic variables are displayed in Fig 4. Left ventricular (LV) catheterization showed gender differences in both the maximum rate of pressure rise (+dP/dt) and the maximum LV relaxation rate (-dP/dt). The Fig 4A shows +dP/dt. The male sham group had a lower +dP/dt than the female sham group (MS: 5140 ± 259 mmHg vs. FS: 6485 ± 178 mmHg). In comparison with the sham groups, orchiectomy (MG = 6728 ± 177 mmHg) and ovariectomy reduced +dP/dt (FG: 5453 ± 229 mmHg). Fig 4B shows-dP/dt. The male sham group had a lower-dP/dt than the female sham group (MS: -3717 ± 129 mmHg vs. FS: -5250 ± 115 mmHg). Gonadectomy increased the-dP/dt in male (MS: -4851 ± 290 mmHg) and decreased-dP/dt in female (FG = -3913 ± 136 mmHg) rats relative to the sham groups.

### Western Blot Analysis

**Fig 5:**
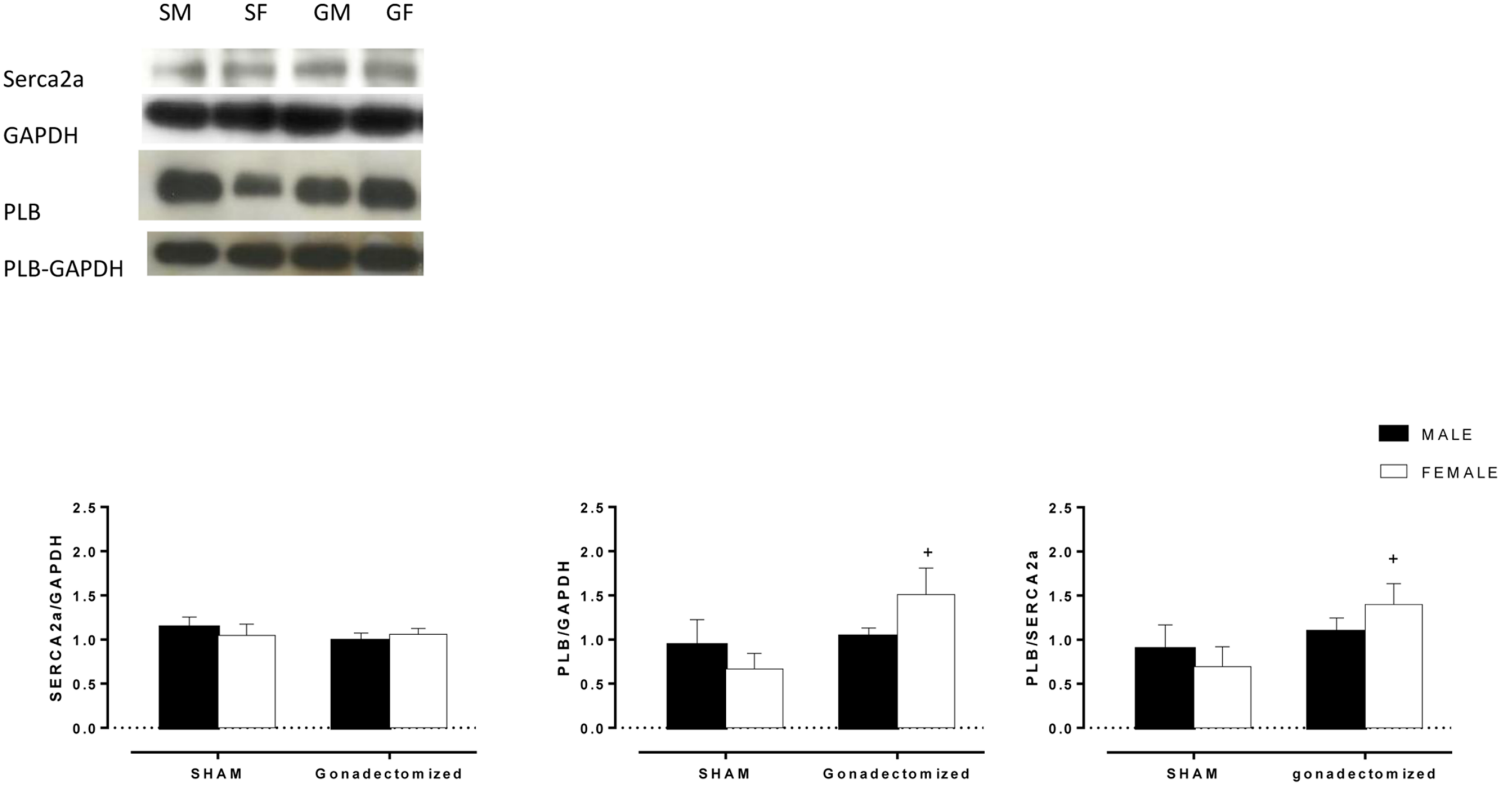
Densitometric analysis of Western blots. (A) SERCA2a; (B) phospholamban (PLB), (C) PLB to SERCA2a ratio, in hearts from male and female sham and gonadectomized rats. (SM: sham male; SF: sham female; GM: gonadectomized male; GF: gonadectomized female). The values are expressed as the means ±S.E.M (n = 6 per group). *P < 0.05 vs. females of the same group; ^+^P < 0.05 vs. sham animals of the same sex (two-way ANOVA and Fischer test).

As shown in Fig 5, we did not find any differences among the groups (male and female) in SERCA2a protein expression (Fig 5A). PLB protein expression (Fig 5B) and the PLB to SERCA2a ratio were increased in female rats after ovariectomy. In males, however, no changes were observed (Fig 5C).

## Discussion

To our knowledge, this is the first study in SHRs that relates hormonal status, cardiac function, trophic effects and the activity of ACEs. Our main findings are that (1) ACE and ACE2 activity were significantly higher in male compared to female rats; (2) orchiectomy decreased the activity of these enzymes while ovariectomy increased ACE2 but did not change ACE activity; (3) gonadectomy improved hemodynamic variables in male rats and worsened them in female rats, (4) In males, changes in contractility were independent of SERCA2a, PLB and the PLB / SERCA2a ratio, while in the females, changes in PLB and the PLB / SERCA2a ratio were observed. Our results suggest that the withdrawal of estrogen and testosterone influences cardiac hypertrophy and the other cardiovascular variables that we evaluated.

Blood pressure was not altered after gonadectomy in both sexes. Similarly, no changes in SBP in female SHRs have been observed after ovariectomy [20,21]. However, a reduction in the blood pressure of male SHR after orchiectomy has been reported [22,23]. These different results could be attributed to methodological differences between the studies, such as: 1) the initial blood pressure was lower, 2) the rats were gonadectomized at a younger age, and 3) SBP was evaluated in anesthetized animals. Thus, in our experiments, the differences in the variables studied between the sham and gonadectomized animals appear to be influenced by sex hormones and not by the SBP, because we observed no change in blood pressure in either sex after gonadectomy.

### Significantly higher ACE and ACE2 activity in males than female rats

The gender is linked to differences in cardiovascular morbidity and mortality [24,25]. Sex hormones may influence the RAS, a system that is linked with these differences [26]. As several authors have demonstrated, testosterone and estrogen directly interact with RAS [3,26,27]. Based on the current literature, classical RAS components are more highly expressed in males and contribute to the development of hypertension [21,23,28], renal and cardiovascular injury [23], as well as changes in the immune system [11]. Conversely, females tend to express more non-classical RAS components such as Ang 1–7. [10,12,29]

In the present study, we observed that in addition to higher ACE activity, the male sham group also showed higher ACE2 activity compared to females. The higher ACE2 activity in male hypertensive animals could be a compensatory mechanism in response to increases in components of the classical arm of RAS that is not observed in female rats. This compensatory mechanism has been reported in many cardiovascular conditions in which an increase in ACE has been reported [30,31]. Burrell et al showed that ACE2 activity increased after myocardial infarction, suggesting that this enzyme plays a part in the negative modulation of RAS in the metabolism of Angiotensin peptides after myocardial injury [30]. Cardiac hypertrophy, fibrosis and hypertension have been found to be associated with an increase in cardiac ACE2 gene expression and ACE2 activity in rats [31]. Thus, ACE2 has been shown to be cardioprotective [32,33]. Study with SHR showed that ACE2 and AT2 gene expression increased in the same direction as classical RAS components, and that cardiovascular hypertrophy was positively associated with increased cardiovascular ACE2 gene expression [34]. In this sense, others reports show increased ACE2 activity in myocardial infarction and cardiac remodeling [35] as a counter-regulatory mechanism that attempts to limit the adverse effects of elevated cardiac Ang II by ACE [36].

### SHR orchiectomy decreases ACE and ACE2 activity while ovariectomy increases ACE2 activity without altering ACE activity

We used gonadectomy as a method to investigate sex differences in the variables studied. We observed that orchiectomy reduced both enzymes and improved variables associated with hypertrophy and hemodynamic function. Conversely, ovariectomy did not alter ACE activity and promoted an increase in ACE2 activity. Furthermore, ovariectomy worsened variables associated with hypertrophy, decreased the number of nuclei, increased heart/tibia length ratios and was associated with a deterioration in hemodynamic functions.

A direct relationship between testosterone and ACE activity has previously been reported [37]. In an earlier study, we demonstrated that male SHRs had higher plasma ACE activity than females and that castration of male SHRs reduced ACE activity. However, the exact mechanisms by which castration in males led to a reduction in ACE and ACE2 in the left ventricle are still the subject of further investigations. There is evidence of reciprocal changes in both enzymes, indicating that ACE2 and ACE may be coexpressed in numerous rat tissues. Rivière et al showed that in Undernutrition-Programmed Hypertension, the activity of ACE or ACE2 was increased in the lung [38]. Animals with hypertension after renal injury and treated with ACE inhibitors (ACEi) showed decreased cardiac ACE and ACE2 activity and an improvement of cardiac structure [31]. Lim et al and us (previous study) demonstrated that castration reduced plasma ACE (-48%) [11,37]. In the present study, male castrated animals had a reduction of 43% in left ventricle-ACE activity. Campbell et al reported marked reductions in the Ang II/Ang I ratio, indicating that ACE is responsible for at least 90% of Ang I conversion to Ang II in blood, kidney, heart, lung, and brain, and at least 77% in the adrenal. Therefore, the reduction in ACE activity influenced the reduction in Ang II. As ACE is the major pathway of formation of Ang II from Ang I, many of the benefits of ACE inhibition can be attributed to a reduction in Ang II levels [39]. Additionally, reducing ACE causes a marked increase in Ang 1–7 levels [40].

Studies in different experimental animal models show estrogen involvement in the regulation of ACE [41–43]. In females SHR, ovariectomy at 19 weeks did not alter plasma ACE activity [11] and in the present work, did not change cardiac ACE activity. Wang et al observed that in the Lewis rat model, neither ovariectomy nor estrogen replacement changed ACE mRNA or activity in the heart [44]. Fabris et al reported that the expression of ACE in the LV was increased in 18 week old SHRs 8 weeks following ovariectomy [45]. We and others have not observed changes in SBP [11,46,47], suggesting that a lack of female hormones may not be the only contributing factor to the elevated blood pressure. In many studies, gonadectomy is conducted soon after weaning, while in some studies, the surgery is conducted in adulthood. Such differences may result in differences in the length of exposure to sex hormones, which in turn may result in differences in sensitivity to hypertensive stimuli [25]. Additionally, the increase of cardiac ACE2 activity in females following ovariectomy is most likely related to hypertrophy of the heart due to the loss of protective estrogen mechanisms in hypertensive rats. It is possible that the increase in cardiac ACE2 activity in ovariectomized female hearts represents an important adaptive mechanism aimed at delaying the progression of adverse cardiac remodeling. There is evidence that ACE2 competes with ACE [48], and AT2 acts as an antagonistic receptor against AT_1_R within RAS [49]. A study by Li et al showed that ACE2 and AT2 gene expression increased in the same direction as classical RAS components [34]. In addition to this unidirectional change of the RAS components, cardiovascular hypertrophy was found to be positively associated with increased cardiovascular ACE2 gene expression.

### Gonadectomy improved the hemodynamic variables in male rats and worsened them in female rats, demonstrating that withdrawal of estrogen and testosterone influences cardiac hypertrophy and other cardiovascular variables

We observed that gonadectomy improved the hemodynamic variables and an index of LV hypertrophy in male rats and worsened these factors in female rats, demonstrating that withdrawal of estrogen and testosterone influences cardiac hypertrophy and other cardiovascular variables. Cardiomyocyte hypertrophy, resulting from an increase in cardiac cell size without cell division, is a strong predictor of cardiovascular morbidity and mortality [50] and occurs in response to mechanical load as well as to neural and humoral factors such as Ang II, endothelin- 1, α1-adrenergic agonists, and peptide growth factors [51]. In our study, ovariectomy increased left ventricular hypertrophy, as measured by the heart weight/tibial length ratio and the number of myocyte nuclei, while gonadectomy reduced left ventricular hypertrophy.

A wealth of evidence indicates that Ang II is a critical factor for stimulating cardiomyocyte hypertrophy, fibroblast proliferation, and extracellular matrix production in LV remodeling [35]. Estrogen is protective against hypertension [13,52], most likely due to amplification of the vasodilator contributions of Ang 1–7 combined with a reduction in the formation and vasoconstrictor actions of Ang II [52] Thus, ovariectomy enhances RAS in SHRs, stimulating RAS in female SHRs [53]. In the male, castration reduces the heart weight [22]. Likewise, Xue et al demonstrated that castration of male mice attenuated the development of AngII-induced hypertension compared with intact males [54]. Furthermore, increases in MAP induced by Ang II were smaller in castrated males than in intact males. Additionally, left ventricular hypertrophy has been shown to lead to a structural rearrangement of the components of the ventricular wall and may result in ventricular stiffening, thereby compromising systolic and diastolic function [55,56]. In our studies, we found differences in cardiac performance between male and female rats. The rates of pressure development (+dP/dt) and decay (-dP/dt) were depressed in male rats compared with females, and gonadectomy improved the hemodynamic variables in male rats while worsening these variables in females. In accordance with Piro et al, gender had a profound impact on cardiac remodeling. Experimental studies suggest that important differences exist between females and males [57]. Ventricular remodeling, characterized by cardiomyocyte hypertrophy, apoptosis, and increased deposition of extracellular matrix proteins, contributing to interstitial and perivascular fibrosis, is among the most dangerous injuries produced by chronic hypertension [58]. This ventricular remodeling could be associated with hemodynamic variables.

### Changes in contractility in males are independent of SERCA2a, PLB and the PLB / SERCA2a ratio, while the females have the participation of the PLB, and PLB / SERCA2a ratio

To investigate whether the changes in hemodynamic variables can be attributed to calcium handling, SERCA2a and PLB expression were compared in male and female rat left ventricles. In agreement with other studies [59,60], we did not find differences in expression of these proteins between male and female sham animals. Hui et al showed significant sex difference in the abundance of Ca^2+^-handling proteins after infarction: calcium L-type channels, ryanodine calcium-release channels (RyR) and Na^+^-Ca^2+^ exchange protein (NCX), but no differences between the sexes in abundance of SERCA2a or PLB were found [59]. In the present work, ovariectomized rats showed increased expression of PLB and a higher PLB to SERCA2a ratio that may have contributed to the reduction in the dP/dt max and min.

Studies in transgenic models suggest that changes in the PLB to SERCA2a ratio is a major regulator of cardiac contractility [61,62]. Moreover, PLB is a repressor of left ventricular basal contractile variables, and alterations in the levels of PLB would be expected to result in alterations in myocardial contractility. Studies using transgenic mice that overexpress cardiac specific PLB suggested that the ‘‘functional stoichiometry” of PLB/SERCA2a is less than 1:1 in native cardiac sarcoplasmic reticulum membranes [63]. In our study, this ratio was two- fold higher in the OVX group compared to the sham group. In male rats, although the expression of SERCA2a, PLB and the PLB to SERCA2a ratio was not changed, orchiectomy reduced the expression of an important component of the RAS system. The observed left ventricle reduction in ACE activity (-43%) could be related to the observed improvement in hypertrophy and cardiac function. Testosterone directly interacts with RAS, upregulating the classical constrictor pathway via upregulation of angiotensinogen gene expression and renin activity [64,65].

## Conclusion

Our data suggest that sex steroids are of great importance for both female and male SHRs in influencing cardiac function and in regulating RAS. In our study, the withdrawal of estrogen in females led to increased cardiac hypertrophy, ACE2 activity, PLB, and PLB to SERCA2a ratio, which could be related to the worsening of hemodynamic variables. In males, the removal of testosterone had the opposite effect on RAS components. The increase in ACE2 activity after ovariectomy and the reduction after orchiectomy seems to be occurring to counterbalance other observed changes. The assessment of sexual dimorphism of the components of RAS can help the continued development of effective therapeutic interventions for cardiovascular diseases.
