# Correction: Sex Hormones Promote Opposite Effects on ACE and ACE2 Activity, Hypertrophy and Cardiac Contractility in Spontaneously Hypertensive Rats

**DOI:** 10.1371/journal.pone.0133225

**Published:** 2015-07-14

**Authors:** EP. L. M. Dalpiaz, A. Z. Lamas, I. F. Caliman, E. H. Ribeiro, G. R. Abreu, M. R. Moyses, T. U. Andrade, S. A. Gouvea, M. F. Alves, A. K. Carmona, N. S. Bissoli

There are two errors in Table 1. The Table legend should say "Left ventricle weight" and the two values in the U/TL column are incorrect. The authors have provided a corrected version of Table 1 here.

**Table 1:** Effect of gonadectomy on body weight (BW), tibial length, uterine weight/body weight (UW/BW) ratio, left ventricle weight (LVW) and weight/tibial length ratio (LV/TL).

Group	Final BW	Tibial Length	U/BW	U/TL	Left Ventricle	LV/TL
n = 7	(g)	(cm)	(mg/g)	(mg/cm)	(mg)	(mg/cm)
SM	278 ± 4*	3.8 ± 0.02*	___	___	763 ± 25*	200 ± 6*
SF	176 ± 2	3.6 ± 0.02	2.3 ± 0.10	110 ± 2.3	545 ± 4,5	150 ± 1
GM	287 ± 9*	3.8 ± 0.02*	___	___	697 ± 11*+	181 ± 3*+
GF	212 ± 2+	3.5 ± 0.04	0.41 ± 0.01+	23 ± 0.6+	602 ± 6+	168 ± 2+

The results are the mean ± S.E.M (n = 6 per group). Variables measured in the Sham(S) and Gonadectomized (G), male (M) and female (F) SHR rats. Statistical significance is indicated by *P, 0.05 vs. females of the same group

+P, 0.05 vs. sham vehicle animals of the same sex. (two-way ANOVA and Fischer test).
